# Organic vs inorganic contribution to the chemistry of cretaceous black shales in the Mamfe basin, SW Cameroon. Evidence from geochemistry and statistical analysis

**DOI:** 10.1016/j.heliyon.2023.e13748

**Published:** 2023-02-15

**Authors:** Bisse Salomon Betrant, Bokanda Ekoko Eric, Florence Njinto Nkwankam, Ashukem Ethel Nkongho, Nanfa Tefak Fatoumata Maelle, Yugye Jules Alex, Belinga Belinga Cedric, Akono Daniel Florent, Nzesseu Nandjou Valentino, Ekomane Emile

**Affiliations:** a School of Geology and Mining Engineering, P.O. BOX 115, Meiganaga, Cameroon; b Department of Geology, University of Buea, Cameroon; c National Higher Polytechnic Institute, University of Bamenda, P.O BOX 039, Bambili, Cameroon; d Department of Earth Sciences, University of Yaoundé I, Cameroon; e Institute of Mining and Geological Research, P.O. BOX 4110, Yaoundé, Cameroon; f National Hydrocarbons Society, Yaoundé, Cameroon

**Keywords:** Mamfe basin, Geochemistry, Cretaceous black shales, Organic components, Inorganic component

## Abstract

The present study focused on evaluating the contribution of both organic and inorganic component to the chemistry of Cretaceous black shales in the Mamfe basin, Sw Cameroon by performing inductively couple plasma spectrometry analyses with the data analysed using multivariate statistical analyses. The shales are classified as calcite enriched (Ca/Mg > 1) and calcite depleted (Ca/Mg < 1). Major elements such as K, Ti, and Al shows significant correlations (>0.71) among themselves and negative correlations with total organic carbon (TOC), total organic nitrogen (TON) and total organic sulphur (TOS) indicating that they were control mainly by inorganic factors. The positive correlation between phosphorus (P) and TOC, TOS, TON maybe due to absorption by organic fraction into the lattice of the shales. Selected both *biophilic* (Ba, Co, Ni, and Sc) and terrigenous (Zr) trace elements were correlated amongst themselves and with organic components. Zr show negative correlation (−0.36) with organic components and correlates negligibly with Co, and Ni (0.04, 0.16) indicating these elements were derived from organic matter with exception to Ba and Sc which shows positive correlation with Zr (0.77) and negatively correlated with TOS (−0.34, −0.13), carbon (−0.25, −0.17) and TON (−0.17, −0.06). The enrichment of light rare elements over heavy rare earth elements positive europium anomaly on PAAS normalise diagrams and an insignificant to negative correlation with TOC, TOS, and TON indicating their derivation from mainly an inorganic factor. Statistical analyses by hierarchical classification ascending (HCA) and principal component analyses (PCA) confirms solely an inorganic contribution to the chemistry of the studied black shales in the Basin. The positive correlations portray by some elements with organic components maybe due to their absorption by organic fractions into their lattice. Further indirect/direct methods such as sequential extraction and FTIR is required throw light on the origin of the chemistry of black shales in the Mamfe basin.

## 1 Introduction

Black shales are thought to be composed of both organic and inorganic components. The inorganic components are related to the weathering of primary crystalline rocks/or from ancient sedimentary rocks while the organic components originated from death remain of plant and animals [1]. In general, researchers [[2], [3], [4], [5]] have loosely analysed the components of the black shales, using the same provenance method as for rocks that contain only clastic components, such as sandstones. Probably, because it is easier to associate the clastic components with crystalline rocks, hence the building block elements of the minerals found in these rocks are of crystalline origin [[6], [7], [8]]. Shales from the Mamfe basin have been considered to compose of pyrite mineral [9] microfossils [10] and organic matter [[11], [12], [13]]. Reference [14] examined some polish shales under SEM (Scanning electron microscopy) and noticed the occurrence of halites of NaCl and gypsum. From the above studies, it is clear that the Cretaceous Mamfe black shales are composed of both organic and inorganic components. The work raised the question of what components contribute to the elemental building block of the shales in the Mamfe basin?. Thus, with the aim of better understanding the chemistry of black shales in the Mamfe, this paper investigates the elemental concentrations which act as the building blocks of black shales from the Mamfe Basin area, South west region, Cameroon. This work takes a holistic approach to determine if the elements forming the building structure of the Mamfe shales were derived from either organic, crystalline or both sources. The approached used in this work will provide a new pathway to sedimentologists all over the world for elements provenance studies of shale rocks as most researchers used element combination and discriminant diagrams for provenance studies laying no emphasis to determine if organic components have influence/alter the composition of the rocks. The information revealed by this work will play a vital role in the understanding of the source composition of the Mamfe shales. The method used in this work will also provide a lead way for researchers working on black shale provenance composition.

## 2 Geology of the study area

**Fig. 1:**
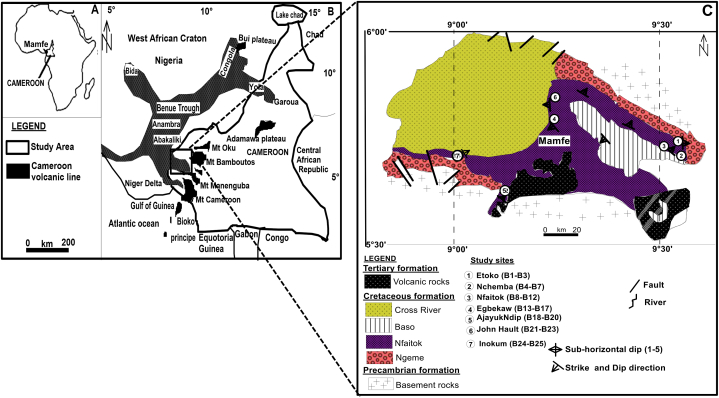
(a) Map of Cameroon in the Africa continent; (b) The Cameroon volcanic line, Mamfe basin and Benue trough in the Cameroon Map; (c) modified geologic map from Ref. [14]of the Mamfe Basin showing the study locations.

The investigated shales (Fig. 1) are found in the Cretaceous sedimentary part of the Mamfe Basin, which is surrounded by Precambrian basement rocks made up of granites, migmatites, syenites, mica-schists, and gneisses [15,16]. Assumed tertiary dykes of volcanic composition penetrate through the sedimentary strata and their underlying Precambrian basement rocks. In the northwestern portion of AjayukeNdip, mafic dykes of basaltic nature are seen cross-cutting coarse-grained sandstones [15,16]. Volcanic rocks of trachytic and phonolitic composition overlay weathered syenites, diorites, and gabbro at Mount Nda Ali in the south-eastern portion of the basin. Reference [15] used microscopic examination of mica-schist in this basin to demonstrate the existence of deformed characteristics including folded muscovite and recrystallized and cracked quartz. Shales, sandstones, conglomerates, and conglomeratic sandstones make up the majority of the sedimentary formations [[12], [13], [14],^17^]. The sandstones are immature, friable to well lithified, and deposited in a river setting [12]. The shales are black to dark grey in colour, deposited in a shallow, oxygenated continental environment [17], while [9] have discovered pyritized carbonaceous shales that are indicative of oxygen deficit conditions. Conglomeratic rocks, which are predominantly found in Etoko and Inokun, are made up matrix and clasts composing of quartzite, granite, gneiss, and schist. Heavy minerals such as zircon, kyanite, rutile, garnet, zoisite, and opaque minerals abound in the sandstones [12]. Zircon grains recovered from alluvial materials yielded Cenozoic, Cretaceous, and Precambrian source rock ages, indicating that they were most likely sourced from the Cameroon volcanic line (volcanic rocks), the Benue Trough, and basement rocks, respectively [15,16,18,19].

Different pyrite morphologies were discovered in the carbonaceous shales (Fc) of the Mamfe Cretaceous Basin (MCB), SW Cameroon, by Ref. [9]. The carbonaceous shales according to these authors, enclose pyritized microfacies, which are made up of laminated (Fcl), oolitic (Fco), marlstone (Fcm), algal mat (Fca), and pelletoidal (Fcp). They argued that the various facies found in the Mamfe basin carbonaceous shales are potential indications of local redox paleoenvironmental settings, displaying evidence of sulphate reduction under saline and carbonate lacustrine setting.

## 3 Methodology

**Fig. 2:**
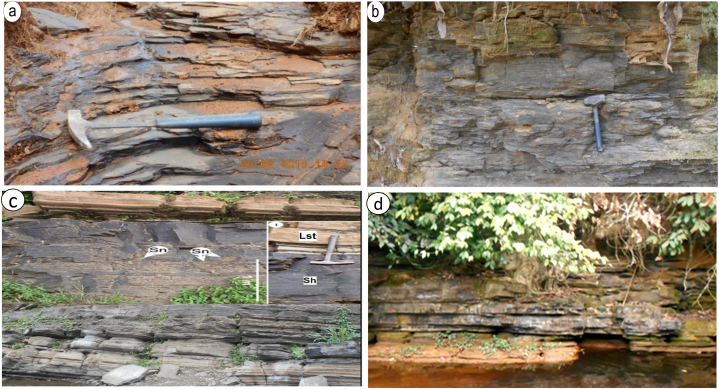
Field photos of Cretaceous black shales from the Mamfe basin. (a) Millimetric to centimetric laminated black shales, (b) massive weathered shales, (c) laminated shales showing nodules of siderite (Sn) interbedding with limestone (lst), (d) centimetric laminated shales.

Amongst the 98 rock samples collected in the field at outcrop level, 45 where shales having variable characteristics in terms of colour and texture (Fig. 2). Amongst the 45 shales collected from the field, 25 were analysed from 07 outcrops, in 07 different sites (S1–S7) as seen in Fig. 1 (coordinates of sites location in Table 1). The choice of the studied outcrop was based on difference in formation as proposed by Ref. [14] and the difference in outcrop angle of dip. The choice in the shales to be analysed from the different outcrops were strictly based on the similarities and differences in their physical properties like texture, colour and their reaction to dilute HCL test (carbonate testing, Fig. 2a and b). Some of the shales show minimal (Fig. 2b) or no reaction with HCl signify a dolomitic property (Fig. 2d).

**Table 1:** Major element oxides (wt%) and total organic nitrogen (TON), total organic carbon (TOC) and total organic sulphur (TOS) data of the studied black shales. Classification (Class.): *Cd = carbonate depleted; Ce = carbonate enriched*.

Study sites/Coordinates	Sample Id	SiO2	AL₂O₃	CaO	MgO	Na₂O	K₂O	Fe₂O₃	MnO	P₂O₅	TiO₂	Ca/Mg	Class.	TON	TOC	TOS
	M1	63.40	16.23	0.48	4.85	2.89	4.20	5.71	0.04	0.19	0.49	0.10	Cd	0.02	0.30	0.06
Site 1: Etoko	M2	63.60	17.64	0.52	3.75	2.92	4.45	4.70	0.04	0.20	0.68	0.14	Cd	0.04	0.79	0.15
5°43′ 13″N, 09°32′09″E	M3	60.80	14.77	5.37	3.41	2.89	3.37	6.81	0.18	0.17	0.70	1.57	Ce	0.04	0.31	0.41
	M4	56.30	17.65	2.98	5.08	1.90	4.56	8.79	0.09	0.19	0.92	0.59	Cd	0.12	0.56	0.09
Site 2: Nchemba	M5	51.60	19.99	3.47	5.60	2.14	4.62	9.74	0.12	0.22	1.00	0.62	Cd	0.07	0.48	0.12
5°42′ 58″N, 09°31′07″E	M6	67.90	12.93	5.59	1.36	4.67	2.57	2.82	0.14	0.24	0.28	4.12	Ce	0.04	0.11	0.07
	M7	61.50	14.10	8.08	1.67	4.86	2.62	4.77	0.23	0.25	0.39	4.85	Ce	0.10	2.34	0.14
	M8	64.60	14.21	6.32	1.49	5.29	2.52	3.17	0.23	0.20	0.44	4.24	Ce	0.03	0.14	0.06
Site 3: Nfaitok	M9	58.30	11.40	12.13	3.76	4.06	3.00	4.91	0.17	0.24	0.53	3.23	Ce	0.06	0.39	0.18
5°43′ 24″N, 09°30′15″E	M10	64.60	13.03	6.66	2.33	5.20	2.82	3.58	0.13	0.25	0.35	2.85	Ce	0.02	0.08	0.08
	M11	48.20	3.12	39.62	2.11	1.83	0.34	2.56	0.26	0.32	0.12	18.82	Ce	0.06	1.44	0.37
	M12	63.90	13.02	9.69	0.81	4.35	1.92	3.73	0.27	0.25	0.57	12.02	Ce	0.17	4.56	0.95
Site 4: Egbekaw	M13	65.00	16.00	3.04	1.56	3.73	3.03	4.93	0.26	0.23	0.76	1.95	Ce	0.18	4.37	0.33
5°41′ 45″N, 09°02′46″E	M14	50.20	6.07	16.87	2.41	2.79	0.56	14.45	3.58	1.27	0.25	6.99	Ce	0.07	2.46	0.62
	M15	60.00	15.81	7.09	1.49	3.78	2.83	4.44	0.51	1.79	0.77	4.76	Ce	0.19	4.83	0.97
	M16	47.80	7.13	32.59	1.47	4.21	0.30	2.76	0.74	1.26	0.26	22.16	Ce	0.05	1.34	0.29
Site 5: AjayukNdip	M17	61.50	12.04	1.32	2.17	0.84	8.49	9.99	0.59	1.01	0.53	0.61	Cd	0.28	12.54	1.43
5°38′ 51.2″N, 09°09′11″E	M18	54.90	10.31	0.66	10.89	0.89	6.70	12.47	0.89	0.20	0.54	0.06	Cd	0.04	0.99	0.25
	M19	63.40	16.94	1.19	2.62	2.94	3.70	6.54	0.04	0.39	0.76	0.46	Cd	0.05	1.21	0.14
Site 6: John Hault	M20	58.0	18.87	0.98	3.50	1.52	4.80	9.36	0.06	0.28	1.12	0.28	Cd	0.08	2.34	0.05
5°45′ 25.6″N, 09°18′59.1″E	M21	51.0	9.94	25.42	2.28	1.64	2.23	4.91	0.09	0.45	0.57	11.14	Ce	0.06	1.88	0.22
	M22	58.2	14.61	0.51	6.61	0.63	7.17	9.47	0.08	0.41	0.77	0.08	Cd	0.09	2.91	0.07
Site 7: Inokun	M23	60.7	16.70	0.57	2.78	0.49	10.78	5.22	0.05	0.29	0.96	0.20	Cd	0.08	1.60	0.04
5°44′ 36″N, 09°01′02″E	M24	59.4	17.14	0.21	0.55	0.81	12.17	7.09	0.12	0.33	0.65	0.38	Cd	0.03	0.34	0.03
	M25	52.7	3.99	23.06	12.68	1.00	0.69	2.50	1.40	0.32	0.16	1.82	Ce	0.03	1.49	0.23

The selected 25 samples of dark grey to black shales (Fig. 2) collected from the field were air dried and powdered at the institute research and geological mining Nkolbison, Cameroon. Sample packaging and preparation was done at the Laboratory of Geoscience of Superficial Formations at the University of Yaoundé 1. The powdered samples were analysed for geochemistry by ICP-AES (inductively coupled plasma – atomic emission spectrometry) and ICP-MS (inductively coupled plasma – mass spectrometry) at the Geological Laboratory of Lakehead University Ontario, Canada. For the analyses regarding ICP-AES and MS, procedures of [[20], [21], [22], [23]]. 0.5 g of the powdered samples was treated with dilute HNO_3_ acid to test the carbonate content of the shales. The samples were dissolved again in an open beaker with concentrated nitric-hydrofluoric acid was added to the samples three times for three days. After digestion, for each sample 2% of nitric double distilled water solution was added to the solution for dilution. For ICP-AES analyses, it was diluted 200 times while for ICP-MS analyses it was diluted 1000 times. A blank was inserted for every ten samples. Accuracy is within 10% and precision 5%.

Based on geochemical data, the shales were classified as carbonate-depleted or carbonate-enriched using their Ca/Mg ratio, as shown in Table 1. In this work, geochemical data for rare earth elements (REEs) were normalized with chondrite composition (see Fig. 2a–d). Anomaly bounds were determined, with >1.05 indicating a positive anomaly, 1.04–0.94 indicating no anomaly, and <0.94 indicating a negative anomaly [3]. The following boundaries were used to determine correlation: from ≥−0.31 to −1.0 = negative correlation; from −0.31 to 0.31 = negligible or no correlation and; >0.31 to 1 = positive correlation [24]. Pearson correlations were based on log (x + 1) transformed data. Correlations marked in red are significant at *p* < 0.05. Correlations were determined with Statistical 13 software.

For determination of total organic carbon (TOC), total organic nitrogen (TON) and total organic sulphur (TOS) analyses, a CARLO ERBA Elemental Analyzer. The samples were loaded into an automated autosampler. When the autosampler is started, the sample is pumped into the combustion reactor, which is maintained at around 1050 °C. The sample container melts in a transient oxygen-rich condition, and the tin promotes a violent reaction (flash combustion). A continuous flow of gas transports the combustion products via an oxidation catalyst of chromium oxide (CrO) stored at 1050 °C within the reaction combustion tube (Helium). To ensure thorough oxidation, a 5 cm layer of silver coated cobalt oxide is put at the bottom of the combustor. The catalyst also traps interfering molecules produced during the combustion of halogenated substances. The mixture of combustion products and water passes through a reduction reactor, which is heated to 650 °C and comprises metallic copper. In the reaction reactor, surplus oxygen is eliminated, and nitrogen oxides from the combustor are decreased to elemental nitrogen at around this temperature, which goes through the absorbent filter with carbon dioxide, sulphur dioxide, and water. C, N, and S, had detection limits of 0.94 g, 0.23 g, and 0.06 g, respectively. Accuracy is within 10% and precision 5%.

## 4 Results

### 4.1 Petrography

**Fig. 3:**
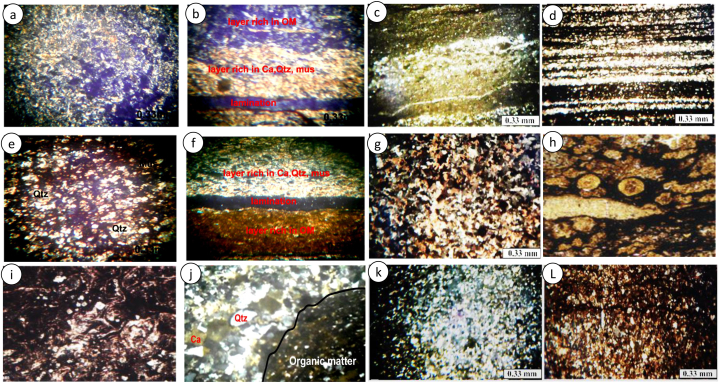
(a–m). Photomicrographs of shales in the Mamfe Basin. (a, h, f, m) scattered minerals mixed with organic matter, (b–d, g) Shales with millimetric to centimetric lamellae composing or organic matter mixed with minerals (i, j) Dolomite-rich algal mat facies; with filamentous calciphytes and phyllodes, (k, l) shales with organic matter occupying an entire domain. Qtz = quartz, Ca = calcite, OM = organic matter, Mus = muscovite.

The shales from acid test show vigorous reactions with dilute HCl while some show little or no reaction at all dilute HCl. The shales display a very fine to silty texture as some of the minerals can easily been identified microscopically. It displays a dark grey colour with laminations (Fig. 3a–l). Most of the quartz grains are very angular to angular grains of in nature. Micas (15–20%) are mostly biotite and muscovite with biotite being oxidised. Organic matter is seen in association with unidentified clay minerals (Fig. 3a–l). Calcites minerals are also seen. The minerals present in the shales display isogranular and laminated clastic microstructure (Fig. 3a–l). The laminations are millimetric to centimetric with lamellae composing of unidentified carbonate minerals, quartz, and organic matter (Fig. 3b–d, g, k). The carbonate minerals in these shales are mostly dolomite and calcite (see SEM images in Refs. [9,25]). The above minerals are bounded together by clayey cement. In samples like Fig. 3b, g, k an entire portion is dominated by organic matter while on other samples the organic matter is scattered randomly missing with clastic materials. Samples Fig. 3i and j are dolomite-bearing algal mat shales. They constitute bioturbated micritic- and organic-rich dolomitic shale which enclose filamentous algal fabrics with crinkled laminations (Fig. 3j) and/or algal calciphytes and phylloids (Fig. 3i), both of which are associated with fish bones and ostracods. Algal mat and peloidal shales show mineralization in dolomite. Algal bounded detrital clastic fragments are common in these shales.

### 4.2 Geochemistry

**Fig. 4:**
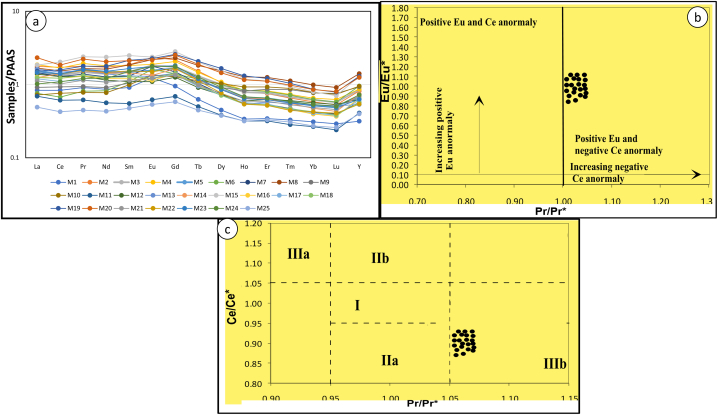
(a) Rare earth element + Y PAAS pattern of whole rock samples (b, c) PAAS normalized cross plot diagram of Ce/Ce* versus Pr/Pr* used as a proxy for the Ce and La anomaly modified from Ref. [12]. Notes: Field I: neither Ce nor La anomaly; Field IIa: positive La anomaly, no Ce anomaly; Field IIb: negative La anomaly, no Ce anomaly; Field IIIa: positive Ce anomaly, negative La anomaly; Field IIIb: negative Ce anomaly.

In the studied shales, SiO_2_ (47.8–67 wt%), Al_2_O_3_ (3.12–16 wt%), and CaO (3–39.62 wt%) show high to moderate content, followed by FeO, MgO, Na_2_O, and K_2_O, while the oxides of Ti, P, and, Mn have concentrations <1.0 wt%. (Table 1). The proportion of TOC in the shale is higher as compared to TOS and TON, while TOS proportion are higher than that of TON (Table 1). Barium shows high content followed by zirconium in the studied shales (Table 3) amongst the studied elements. Rare earth element data of the studied shales are presented on Table 5 and their PAAS normalized characteristics seen in Fig. 4a–c and Table 5 display: an enrichment of middle rare earth elements over heavy rare earth elements, a positive Eu anomaly (Fig. 4a–c); borderline negative Ce anomaly (Fig. 4b); and a positive Gd anomaly. The total rare earth elements (REE) content of the studied shale varies from 92 to 414 mg kg^−1^, with a mean value of 233.5 mg kg^−1^. The average value of REEs of the shales are greater than the mean value of the North American Shale Composite (NASC) (167.41 mg kg^−1^), world-wide black shales (134.19 mg kg^−1^). The LREE/HREE ratios of the studied shale vary from 5.8 to 30.8 with an average of 18.3.

**Table 2:** Major element correlation with total organic nitrogen (TON), total organic carbon (TOC), and total organic sulphur (TOS) components for the Mamfe black shales. Bold values are significant correlations for (at p < 0.05).

Variables	SiO2	AL₂O₃	CaO	MgO	Na₂O	K₂O	Fe₂O₃	MnO	P₂O₅	TiO₂	TON	TOC	TOS
SiO2	1												
AL₂O₃	0.541	1											
CaO	−0.700	−0.815	1										
MgO	−0.231	0.153	−0.342	1									
Na₂O	0.478	0.029	0.027	−0.565	1								
K₂O	0.293	0.505	−0.724	0.558	−0.546	1							
Fe₂O₃	−0.318	0.068	−0.339	0.590	−0.650	0.486	1						
MnO	−0.405	−0.508	0.216	0.013	−0.100	−0.257	0.595	1					
P₂O₅	−0.334	−0.321	0.294	−0.311	−0.021	−0.186	0.139	0.542	1				
TiO₂	0.130	0.828	−0.597	0.289	−0.360	0.512	0.322	−0.358	−0.169	1			
TON	0.125	0.092	−0.157	−0.286	−0.202	0.343	0.143	0.051	0.424	0.238	1		
TOC	0.087	−0.074	−0.097	−0.275	−0.299	0.412	0.210	0.153	0.475	0.055	0.912	1	
TOS	−0.016	−0.243	0.049	−0.276	−0.206	0.193	0.183	0.318	0.616	−0.083	0.817	0.875	1

Values in bold = significant.

**Table 3:** Biophiles and lithophile trace element concentrations (mg kg^−1^) and organic components (TON, TOC, TOS in ppm) of the Mamfe studied black shales.

Concentrations	M1	M2	M3	M4	M5	M6	M7	M8	M9	M10	M11	M12	M13
Ba	1470	1281	1534	981	1003	978	1794	1156	916	851	132	407	745
Co	16	20	27	37	42	18	16	14	24	19	4	23	18
Ni	27	33	42	73	66	18	33	24	30	17	11	32	32
Sc	9	13	14	20	23	7	8	12	10	9	3	11	12
Zr	131	107	76	90	86	66	93	68	102	77	3	8	6
TON	200	400	400	1200	700	400	1000	300	600	200	600	1700	1800
TOC	3000	7900	3100	5600	4800	1100	23,400	1400	3900	800	14,400	45,600	43,700
TOS	600	1500	4100	900	1200	700	1400	600	1800	800	3700	9500	3300

	M14	M15	M16	M17	M18	M19	M20	M21	M22	M23	M24	M25
Ba	136	420	144	704	502	764	712	352	542	834	813	347
Co	13	34	8	75	28	23	34	18	47	33	47	6
Ni	30	54	21	105	37	54	75	48	53	50	54	12
Sc	8	15	7	10	11	16	25	12	12	15	14	4
Zr	4	6	4	4	3	3	9	2	76	95	81	1
TON	700	1900	500	2800	400	500	800	600	900	800	300	300
TOC	24,600	48,300	13,400	125,400	9900	12,100	23,400	18,800	29,100	16,000	3400	14,900
TOS	6200	9700	2900	14,300	2500	1400	500	2200	700	400	300	2300

**Table 4:** Biophile trace element correlation with total organic nitrogen (TON), total organic carbon (TOC), and total organic sulphur (TOS) components for the Mamfe black shales. Bold values are significant correlations for (at p < 0.05).

Variables	Ba	Co	Ni	Sc	Zr	TON	TOC	TOS
Ba	1							
Co	0.094	1						
Ni	0.072	0.888	1					
Sc	0.223	0.498	0.691	1				
Zr	0.773	0.100	−0.041	0.160	1			
TON	−0.171	0.563	0.576	0.132	−0.360	1		
TOC	−0.250	0.579	0.554	−0.056	−0.458	0.906	1	
TOS	−0.341	0.369	0.330	−0.169	−0.519	0.797	0.851	1

Values in bold = significant.

**Table 5:** Rare earth element contents (mg kg^−1^) in shale samples and associated geochemical parameters. Legend: L/H = LREE/HREE; L/M = LREE/MREE; M/H = MREE/HREE; Ce/Ce* = Ce_*n*_*/(La* × *Pr)*_*n*_^*0.5*^; Eu/Eu* = Eu_*n*_*/(Sm × Gd)*_*n*_^*0.5*^.

Sample ID	La	Ce	Pr	Nd	Sm	Eu	Gd	Tb	Dy	Ho	Er	Tm	Yb	Lu	Y	∑REE	∑(L/H)	∑L/∑M	∑M/∑H	Ce/Ce*	E/E*
M1	31.88	66.52	8.02	29.2	5.05	1.34	4.44	0.49	2.1	0.33	0.98	0.13	0.87	0.13	8.63	13.45	8.4	1.11	1.16	0.87	1.82
M2	67.67	136.59	15.81	58.53	10.35	2.54	10.15	1.18	4.99	0.78	2.14	0.26	1.64	0.23	22.32	18.46	5.97	1.1	1.09	0.88	1.75
M3	53.17	106.19	13.08	47.43	8.27	1.98	8.11	1.01	4.67	0.78	2.19	0.28	1.75	0.25	23.25	228.98	12.84	1.11	0.88	1.04	1.43
M4	71.1	134.15	17.13	61.2	10.08	2.04	9.62	1.14	5.06	0.83	2.34	0.3	1.84	0.27	23.58	135.02	14.89	1.26	0.93	1.02	1.7
M5	59.46	109.52	14.23	50.37	8.26	1.68	7.75	0.91	4.04	0.66	1.85	0.24	1.48	0.22	18.54	151.05	14.41	1.28	0.95	1.03	1.38
M6	28.96	53.71	7.16	27.89	6.12	1.74	6.55	0.92	4.67	0.83	2.29	0.29	1.76	0.25	26.07	393.77	12.26	0.74	0.72	0.88	1.13
M7	55.38	102.26	12.25	43.25	7.26	1.92	6.95	0.8	3.47	0.55	1.53	0.19	1.18	0.17	15.58	274.14	30.77	1.18	1.1	1.02	1.22
M8	63.99	122.36	14.61	55.12	10.29	2.37	10.78	1.41	7.18	1.26	3.57	0.45	2.79	0.39	38.42	717.89	13.58	1.02	0.71	1.03	1.84
M9	34.87	74.06	8.28	30.84	5.83	1.46	6	0.83	4.27	0.81	2.45	0.34	2.17	0.33	25.4	300.76	17.5	1	0.61	0.93	1.06
M10	27.6	60.56	6.88	26.25	5.51	1.45	5.94	0.87	4.82	0.92	2.64	0.36	2.2	0.32	25.88	376.51	11.41	0.84	0.56	0.95	1.05
M11	26.48	48.61	5.44	19.1	3.05	0.67	3.2	0.39	1.79	0.31	0.91	0.11	0.75	0.1	11.14	302.53	23.03	1.34	0.8	0.85	1.53
M12	53.07	101.93	11.48	38.87	6.07	1.18	5.85	0.7	3.38	0.59	1.79	0.24	1.59	0.23	17.26	129.67	17.71	1.49	0.76	1.03	1.05
M13	62.28	117.42	13.74	47.24	7.29	1.4	6.66	0.75	3.33	0.54	1.64	0.22	1.49	0.22	14.57	70.06	15.45	1.5	0.92	1.04	1.2
M14	47.74	111.25	13.67	48.77	8	1.45	7.58	0.91	4	0.65	1.81	0.22	1.34	0.19	20.24	425.05	22.97	1.28	0.92	1.02	1.35
M15	70.48	161.71	21.14	80.33	13.82	2.56	13.16	1.57	7.22	1.17	3.19	0.38	2.24	0.31	35.32	92.55	19.03	1.12	0.92	0.82	2.34
M16	48.48	96.75	11.5	41.04	6.66	1.23	6.52	0.75	3.33	0.54	1.52	0.18	1.12	0.16	17.9	360.84	19.69	1.34	0.94	1.09	1.12
M17	45.72	87.85	10.18	36.8	6.34	1.32	6.38	0.73	3.33	0.56	1.55	0.19	1.15	0.16	16.91	55.89	15.39	1.22	0.93	1.08	1.08
M18	47.46	97.03	11.74	42.23	7.09	1.32	6.64	0.77	3.32	0.54	1.5	0.19	1.2	0.17	15.55	53.03	6.89	1.28	0.97	1.02	1.18
M19	58.21	126.13	15.73	60.07	11.89	2.45	12.04	1.6	7.75	1.3	3.46	0.42	2.43	0.34	35.44	43.64	7.34	0.95	0.78	1.05	2.12
M20	88.81	146.79	19.67	69.57	11.74	2.34	11.76	1.46	6.76	1.15	3.16	0.4	2.41	0.35	34.14	17.41	5.77	1.24	0.82	0.98	2.04
M21	42.77	81.39	10.11	36.49	6.33	1.31	6.28	0.76	3.54	0.59	1.69	0.21	1.37	0.2	18.9	315.58	13.44	1.18	0.84	1.03	1.09
M22	55.8	110.62	13.75	49.77	8.5	1.61	7.8	0.87	3.58	0.53	1.48	0.18	1.19	0.18	14.75	34.31	9.76	1.25	1.11	1.01	1.4
M23	55.67	111.86	14.05	51.54	9.18	1.9	8.44	0.93	3.98	0.64	1.77	0.23	1.44	0.21	17.15	18.47	7.17	1.14	1.07	1.02	1.5
M24	38.86	86.55	10.71	41.38	8.34	1.92	8.19	0.98	4.31	0.68	1.86	0.24	1.5	0.22	19.78	36.18	6.49	0.9	0.98	1.02	1.42
M25	18.73	33.88	3.95	14.64	2.64	0.58	2.71	0.35	1.77	0.31	0.94	0.12	0.77	0.11	10.85	157.91	12.05	1.13	0.69	0.87	1.47

### 4.3 Multivariate statistical analyses

**Fig. 5:**
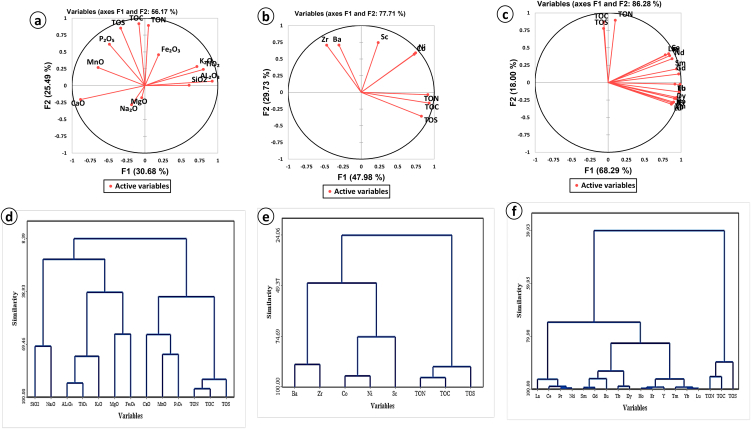
Statistical analyses diagram (a) PCA for major elements oxides, (b) PCA for trace elements, (c) PCA for rare earth elements (REEs), (d) HCA for major elements oxides, (b) HCA for trace elements, (c) CCA for rare earth elements (REEs).

Negative correlations are observed between: TOS and MgO (r = −0.25); TOC and MnO (r = −0.28); TOC and MgO (r = −0.16). Amongst the major elements, negative correlations exist between MnO and SiO_2_ (r = −0.47), SiO_2_ and Al_2_O_3_ (r = −0.62), SiO_2_ and K_2_O (r = −0.49), SiO_2_ and TiO (r = −0.52) (Table 2). Fig. 5a, d shows no correlations of major elements oxides with organic components. The concentration of the biophiles elements in the studied shales (Table 3) were correlated with terrigenous zirconium alongside organic component carbon (TOC), nitrogen (TON) and sulphur (TOS). Ba correlates positively with Zr (0.77), and negative correlation with TOC (−0.25), TON (−0.17) and TOS (−0.34). Co and Ni which correlates positively (0.89) as seen in Table 4 showing an insignificant to negative correlation with zirconium (0.10 and −0.04 respectively). These biophile elements correlates positively with TOC and TON (r > 0.5; Table 4). Scandium shows no correlation with zirconium (0.16) but correlates negatively with TOC and TOS (Table 4). The PCA and HCA analyses show no relationship between the selected biophilic trace elements with the organic components (Fig. 5b, e).

PCA (Principal component analyses) was performed using the normalized values (Fig. 5a–c). HCA (Hierarchical Classification Ascending) between the different element (major, trace and rare earth elements) against TOC, TON and TOS group main and sub-clusters (Fig. 5). For the major elements, two main clusters are seen with oxides of Si, Na, Al, Ti, K, Mg and Fe in one cluster and oxides of Ca, Mn, P, TON, TOS, and TOC on the other cluster. The first cluster further divides in to two subclusters with one ending with oxides of Si and Na while the other clusters further divide in to two other smaller clusters ending with oxides of Al and Ti and the other ending with oxides of Mg, Fe and K. The second main cluster divides in to two sub cluster with one ending with oxides of Ca, Mn and P and the other with ending with TOS, TOC and TON (Fig. 5d). For the trace elements two main cluster were also seen with one cluster ending with all the analysed trace elements and the other with the TON, TOS and TOC (Fig. 5e). With the trace elements some sub cluster shows ending of Ba and Zr, Co and Ni while Sc was isolated (Fig. 5e). The REEs clustered alongside TON, TOS and TOC shows similar pattern as that of trace elements where the two principal cluster consist of the entire REEs and component of TOC, TON and TOS (Fig. 5c, f).

## 5 Discussion

### 5.1 Major element distribution and controlling factors

**Fig. 6:**
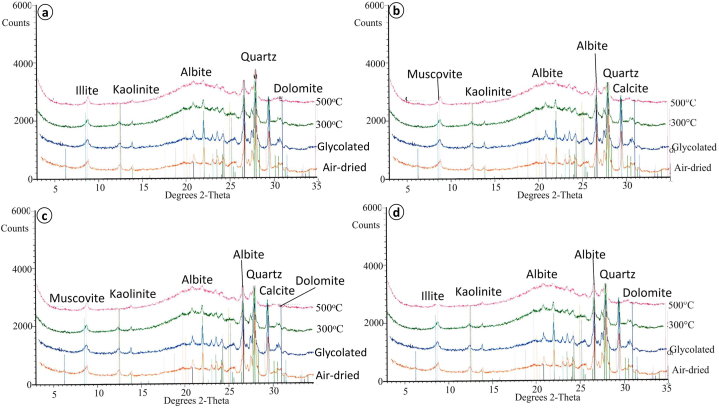
Xrd spectral lines for the studied black shales in the Mamfe basin. (a, c, d) dolomitic shales, (b) carbonated shales.

According [26], organic sulphur (TOS) in shales and coal are form as a result of the reaction of amino acid sulphur (H_2_S) source from plant with organic matter. The TOS content agrees with the presence of pyrite and organic matter in the studied shales, as noted by Refs. [9,11]. Some of the shales have Ca/Mg ratio >1 indicating that they are calcite enriched, while some samples are considered calcite depleted and dolomitic due to their Ca/Mg ratio<1. The carbonaceous and dolomitic aspect the shales in the Mamfe basin was also revealed by works published by Refs. [9,25] and Xrd mineralogy (Fig. 6a–d). Quartz and clay minerals are primarily related to oxides of Si, Al, Ti, and K. The positive correlations among these elements (Table 2) show that SiO_2_, Al_2_O_3_, TiO, and K_2_O are primarily derived from a mixed clay assemblage, which is indicative of the presence of kaolinite, illite, and illite/smectite mixed layers as revealed in Fig. 4 though SiO_2_ in the shales could originate from mixed proportions of following origins: hydrothermal, detrital, and biogenetic sources [7]. The insignificant association between Fe and TOS, shows that some sulphur in the shales is stored in the form of pyrite. The insignificant correlation between TOS, TOC and Fe_2_O_3_ contents (Table 2) indicates that Fe is not present in organic matter.

No correlation is seen between CaO and TON and TOS (Table 2). This lack of correlation may imply Ca presence in more than one form [27]. Apart from the mineralogical evidence of Ca presence in the shales, the Ca content may also be related to carbonate shell fossils. Oxide of P show strong significant positive correlation with TOS (0.47) and TOC (0.6) from Pearson's correlation matrix (Table 2) and falls in the same quadrants as TOS and TOC (Fig. 5a) but show no cluster ending relationship (Fig. 5d). Reference [28] observed that, the compositions of fossil shells, common in shale, are nearly pure Ca-carbonate, but some include detectable traces of P, suggesting that P is partly present in fossil shells. This point is supported by research carried out by Ref. [9] in the Mamfe Basin that revealed carbonaceous shales with bones and faecal pellets, both probed under SEM (Scanning electron microscopy) to compose of Ca_3_(PO_4_)_2_, which stands as the possible sources of P. In addition, the insignificant relationship (r = 0.30) between CaO and P_2_O_5_ contents in the shales discard the influence of phosphate content in the shale [29]. The insignificant correlation between Na_2_O and CaO discard their association to pore water [30,31] and silicate minerals [32,33].

Manganese (Mn) display strong carbonate affinity in organic fraction [34,35], and show an organic affinity in low-rank or low-ash coals [36]. But in the case of the Mamfe shales there is insignificant correlation between CaO and MnO (r = 0.21) which is far less compared to that found within organic fractions. This insignificant relationship discards an organic contribution to the chemistry of the studied shales. Generally, the HCA analyses shows no relationship between the major elements and the organic fractions (Fig. 5d).

### 5.2 Trace element distribution and controlling factors

Most of the trace elements found in the black shales may come from various sources. These sources can either be organic (coming from remains of plant and animals), terrigenous (coming from pre-existing rocks) and diagenesis (transformation, recrystallization and precipitation). For zones devoid of mining activities and urban development which may contaminate the shales and alter the trace elements content, their origin can be derived from biogenic and non-biogenic sources. Trace elements of organic sensitivity known as biophiles have been used to determine the sources of organic matter in black shales [13,37]. These elements (Ba, Co, Ni, and Sc) are mostly found associated in organic matter through syngenetic adsorption and complexation by humic acids [38] and hydrothermal activity during organic matter transformation [1,39]. The concentration of the biophiles elements in the shales of the Mamfe shales were correlated with terrigenous zirconium alongside organic component such as TOC, TON and TOS to trace if their origin is the same. The positive correlation of Ba with Zr and negative correlation with organic components shows that Ba in the Mamfe shales were derived from an inorganic source with no organic influence. Cobalt (Co), and nickel (Ni), are naturally magnetic. These elements are strongly associate with organic fractions [40]. Co and Ni correlates positively between themselves but show no or negligible correlation with zirconium and correlates positively with TOC and TON (Table 4) indicating that their origin may be traced from organic components forming the Mamfe shales. Scandium correlates negatively with TOC and TOS (see Table 4) indicating that they were contributed from an inorganic source. The discrepancy in the correlation among the trace elements proves an inorganic and organic source for the trace element in the Mamfe shales. The conclusive assumption of the contribution of organic and inorganic contribution to the chemistry of the Mamfe shales through Pearson's correlations may seem inconsistence since some of the correlations maybe false. The PCA and Cluster (Fig. 5a and b) analyses employed to clear doubts on the results of Pearson's correlation shows that non the trace elements were sourced from organic fractions as none falls within the quadrants and branch of organic fractions.

### 5.3 REEs distribution, controlling factors

Generally, in shales, REEs distribution show an enrichment of light rare earth elements (LREEs) over heavy rare earth elements (HREEs) [29,[41], [42], [43]]. The LREE/HREE ratios of the shale samples from the Mamfe basin vary from 5.8 to 30.8 with an average of 18.3. This ratio for the studied shales is twice greater than that of river oil shale (7.8 mg kg^−1^) studied by Ref. [44]. All samples from the Mamfe basin show a negative Eu anomaly and a negligible Ce anomaly which is similar to the river oil shale samples (Eu* = 0.62–0.71; Ce* = 0.91–0.97) studied by Ref. [44] in the Northern Tibet, China, which are thought to derive their rare earth signatures from an inorganic source. The high Eu anomaly in PAAS-normalized diagram (Fig. 3a–c), indicates that REEs of the Mamfe shales may have been derived from an inorganic source mainly crystalline rock [45].

[46] studied the geochemical elements variations of kerogen (organic fraction) extracted from black shales in the early Cambrian Formation in Guizhou province, South China. They grouped the rare earth elements into four groups based on their REE elements characteristics. To them, organic matter characterised by minor enrichments of light rare earth element (LREE) with modest PAAS negative Ce anomalies falls under group I. Modest enrichments of middle rare earth element (MREE) with modest to strong PAAS negative Ce anomalies organic fractions were grouped as II. The organic samples composed of strong depletions of heavy rare earth element (HREE) with weak or no negative PAAS Ce anomalies were place as group III, while samples with minor enrichments of HREE with almost no significant Ce anomaly were placed as group IV. The studied shale samples on PAAS characteristic display a strong enrichment of light rare earth elements over heavy rare earth elements (Table 5), low middle rare earth elements over heavy rare earth elements (Table 5), and a negative to no Ce anomaly (Fig. 3b and c). The dissimilarities of REEs PAAS characteristics of the organic fraction studied by Ref. [46] in the black shale of the Guizhou province formation to the shales of the Mamfe basin discard any influence/contribution of organic fraction to the REEs composition. The above conclusion may be right but is weak in the sense that rare earth elements in organic fractions may differ from one geographical location to another. In addition, no standard parameter has been put forward were REEs in organic fraction can be normalized, except for PAAS. With such limitation to assess the contributions of both organic and inorganic fractions to black shales chemistry, this work developed a correlation approach of REEs and organic fractions to overcome such barrier. Individual rare earth elements were correlated with organic fraction. Table 6, proves that as all the REEs were either negatively correlated or show insignificant correlations with organic components of TOS, TON and TOC indicating that these elements are not related with organic association. The strong positive correlations which exist among the elements in the REE group show that only the inorganic fraction is responsible for their presence in the Mamfe shales. The PCA and cluster analysis confirms the contribution of inorganic components to the rare earth elements chemistry of the black shales in the Mamfe basin.

**Table 6:** Correlation of rare earth elements with organic components (TON, TOC, and TOS). Bold values are significant correlations for (at p < 0.05).

	La	Ce	Pr	Nd	Sm	Eu	Gd	Tb	Dy	Ho	Er	Tm	Yb	Lu	Y	TON	TOC	TOS
La	1.00																	
Ce	0.98	1.00																
Pr	0.97	0.99	1.00															
Nd	0.96	0.98	1.00	1.00														
Sm	0.88	0.93	0.95	0.97	1.00													
Eu	0.75	0.79	0.81	0.85	0.93	1.00												
Gd	0.85	0.89	0.91	0.94	0.99	0.95	1.00											
Tb	0.74	0.78	0.81	0.85	0.93	0.93	0.97	1.00										
Dy	0.65	0.69	0.72	0.76	0.87	0.89	0.92	0.98	1.00									
Ho	0.54	0.58	0.60	0.65	0.77	0.81	0.84	0.94	0.98	1.00								
Er	0.54	0.58	0.60	0.64	0.75	0.80	0.82	0.92	0.98	0.99	1.00							
Tm	0.48	0.51	0.52	0.57	0.69	0.75	0.76	0.88	0.94	0.98	0.99	1.00						
Yb	0.50	0.54	0.54	0.59	0.70	0.75	0.76	0.86	0.93	0.96	0.98	0.99	1.00					
Lu	0.47	0.50	0.51	0.55	0.66	0.72	0.73	0.83	0.91	0.95	0.97	0.99	1.00	1.00				
Y	0.50	0.53	0.56	0.60	0.72	0.75	0.80	0.90	0.96	0.98	0.98	0.96	0.94	0.92	1.00			
TON	0.36	0.34	0.31	0.28	0.18	0.02	0.15	0.06	0.03	−0.01	0.01	−0.03	−0.01	−0.04	0.00	1.00		
TOC	0.17	0.16	0.13	0.10	0.02	−0.13	0.00	−0.08	−0.10	−0.13	−0.13	−0.17	−0.17	−0.20	−0.11	0.91	1.00	
TOS	0.07	0.11	0.07	0.04	−0.04	−0.18	−0.04	−0.08	−0.08	−0.08	−0.08	−0.11	−0.12	−0.16	−0.03	0.80	0.85	1.00

Values in bold = significant.

## 6 Conclusion

The Cretaceous black shales in the Mamfe basin were investigated geochemically to revealed their composition, classification and the contribution of both organic and inorganic component to their chemistry. This work employed the indirect method of Statistical analysis.

Correlation coefficients Factor analysis (PCA) and Cluster analysis (HCA) though some statistical results may deviate from the geological fact. Based on classification, the shales of the Mamfe Basin were divided into two categories based on their Ca/Mg ratio as calcite enriched shales (carbonaceous shales) with Ca/Mg ratio >1 and calcite depleted shales or dolomitic shales Ca/Mg ratio <1 as also revealed by Xrd mineralogy. The organic components of the shales such as TOC, TON and TOS have different variations with the proportion of TOC > TOS > TON which were correlated with major, trace and REEs in the shales. The major elements oxides of Si, Ti, Mn, Fe, Mg, K, Na and Ti shows insignificant correlations from Pearson's coefficient and PCA with exception to P which show positive correlation with TOC, TOS and TON. The selected biophilic trace element indicates inorganic source for barium and scandium while Co and Ni show an inorganic derivation. Rare earth elements in the shale have originated from an inorganic source due to the negative correlation with TOC, TOS and TON as revealed by Pearson's coefficients, PCA and cluster analysis. In essence, the majority of the studied elements were derived from an inorganic source, though one cannot neglect the minute contribution of the absorption some biophile trace elements and oxide of P in to the organic lattice provoking the positive relationship.

This work is the first of its kind in the area. It has provided evidence on both the quantification of element and their sources (organic or inorganic). However, this study still has drawbacks that should be clarified for better understanding of the exact sources of the elements forming the cretaceous back shales. Indeed, it was based on indirect statistical methods, which may have some mathematical errors and deviate from geological facts. Thus, other indirect methods such as physical separation and sequential extraction coupled with direct methods such as electron beam ion method and spectrometry (FTIR) speciation studies should be carried out.

## Author contribution statement

Dr Bisse Salomon Bertrant, Dr Bokanda Ekoko Eric, & Ashukem Ethel: Conceivedand designed the experiments; Performed the experiments; Wrote the paper.

Dr Yugye Jules Alex, Ms Nanfa Tefak Fatoumata Maelle & Mr Belinga Belinga Cedric: Analysed and interpreted the data; Wrote the paper.

Professor Ekomane Emile, Dr Florence Njinto Kwankam, Akono Daniel & Nzesseu Nandjou Valentino: Contributed reagents, materials, analysis tools; Wrote the paper.

## Funding statement

Analytical work was supported by a Discouvery Grant to PWF from the Natural Sciences and Engineering Council of Canada under the supervision of Prof Fralick in the Geological Laboratory of Lakehead University, Ontario Canada.

## Data availability statement

No data was used for the research described in the article.

## Declarations of interest's statement

The authors declare that they have no known competing financial interests or personal relationships that could have appeared to influence the work reported in this paper.
